# Cellular heterogeneity in metabolism and associated microbiome of a non-model phytoflagellate

**DOI:** 10.1093/ismejo/wraf046

**Published:** 2025-03-09

**Authors:** Aditya Jeevannavar, Javier Florenza, Anna-Maria Divne, Manu Tamminen, Stefan Bertilsson

**Affiliations:** Department of Biology, University of Turku, 20500 Turku, Finland; Department of Ecology and Genetics, Uppsala University, 752 36 Uppsala, Sweden; Department of Organismal Biology, Uppsala University, 752 36 Uppsala, Sweden; Science for Life Laboratory, Department of Cell and Molecular Biology, Uppsala University, 752 37 Uppsala, Sweden; Department of Biology, University of Turku, 20500 Turku, Finland; Department of Aquatic Sciences and Assessment and Science for Life Laboratory, Swedish University of Agricultural Sciences, 756 51 Uppsala, Sweden

**Keywords:** single-cell transcriptomics, smart-seq2, *Ochromonas triangulata* SSU rRNA, uncharacterized diversity, microeukaryote, bacterial community

## Abstract

Single-cell transcriptomics is a key tool for unravelling metabolism and tissue diversity in model organisms. Its potential for elucidating the ecological roles of microeukaryotes, especially non-model ones, remains largely unexplored. This study employed the Smart-seq2 protocol on *Ochromonas triangulata*, a microeukaryote lacking a reference genome, showcasing how transcriptional states align with two distinct growth phases: a fast-growing phase and a slow-growing phase. Besides the two expected expression clusters, each corresponding to either growth phase, a third transcriptional state was identified across both growth phases. Metabolic mapping revealed a boost of photosynthetic activity in the fast growth over the slow growth stage, as well as downregulation trend in pathways associated with ribosome functioning, CO_2_ fixation, and carbohydrate catabolism characteristic of the third transcriptional state. In addition, carry-over rRNA reads recapitulated the taxonomic identity of the target while revealing distinct bacterial communities, in co-culture with the eukaryote, each associated with distinct transcriptional states. This study underscores single-cell transcriptomics as a powerful tool for characterizing metabolic states in microeukaryotes without a reference genome, offering insights into unknown physiological states and individual-level interactions with different bacterial taxa. This approach holds broad applicability to describe the ecological roles of environmental microeukaryotes, culture-free, and reference-free, surpassing alternative methods like metagenomics or metatranscriptomics.

## Introduction

Microbial eukaryotes comprise the vast majority of known eukaryotic lifeforms [1]. They showcase a plethora of lifestyles, from free-living to strictly parasitic [2], photosynthetic, phagotrophic, or both [3, 4]. They exhibit a varied catalogue of gene expression quirks, such as mRNA fragmentation, *trans*-splicing, or translational slippage [5]. They are ubiquitous across most aquatic and terrestrial environments [6, 7], and are subject to very strong spatial and seasonal constraints [8, 9]. The unifying feature that groups them together, beyond their eukaryotic characteristics, is that they are single-celled and, therefore, microscopic. Their small size, coupled to the difficulty of culturing the overwhelming majority of known single-celled eukaryotic organisms [10], has technically limited the study of their taxonomy, physiology, and ecology to those species that could be readily cultured or visually recognized under the microscope.

The advent and subsequent development of techniques based on nucleic acid sequencing has circumvented these limitations, greatly expanding our understanding of microeukaryote biology [11, 12]. During the last two decades, several *en masse* approaches based on molecular characterization (i.e. “omics” methods) have developed into pivotal tools to study the composition and functional potential of microbial communities. Many of these approaches, however, lack single-cell resolution and therefore cannot capture heterogeneity at the organismal level. Single-cell methods have emerged to overcome this limitation. The advantages of targeting individual cells are multiple: assays require little starting material, are independent of culture availability, are not limited by high microbial community diversity, and provide resolution at organismal level. In the case of single-cell mRNA sequencing (scRNA-seq), single-cell resolution allows accessing transcriptomic information that would otherwise be too scarce to be recovered from bulk extraction (e.g. when the abundance of target organisms is low), and can resolve coexisting, conspecific cell types with disparate gene expression profiles which otherwise would be averaged in an integrated sample.

Despite the potential, studies targeting gene expression in microeukaryotes at single-cell levels are scarce, and mostly committed to either well-studied model organisms [13–15] or pathogenic apicomplexans, such as *Plasmodium* [16–18] and *Toxoplasma* [19]. To date, only three publications exist that offer insight into microeukaryote gene expression beyond either of these lineages: one exploring the feasibility of expression-targeted scRNA-seq in two small flagellates, the haptophyte *Prymnesium parvum* and the dinoflagellate *Karlodinium veneficum* [20], and two other uncovering the dynamics of infection of specific giant viruses within their eukaryotic hosts [21, 22]. This paucity of studies is partly a consequence of methodological constraints and challenges associated with single-cell transcriptomic techniques, such as the need for rapid cell isolation upon sampling, low throughput of isolation strategies compatible with large cells where manual cell isolation is the only feasible option, the low number of mRNA molecules per individual cells or the difficulty of lysing walled or shielded cell types [20, 23]. Despite these limitations, the *Plasmodium* and *Gephyrocapsa* (formerly *Emiliania*) *huxleyi* examples showcase scRNA-seq as a feasible approach to metabolically map distinct cell stages within complex microbial populations. However, both examples rely on the availability of either good reference genomes or an integrated reference transcriptome to which scRNA-seq reads can be mapped to build single-cell expression profiles. Given the difficulty of culturing most of the enormous diversity of single-celled eukaryotic life forms, such resources are typically scarce. In these cases, an *ad hoc*, partial transcriptomic reference can still be built by assembling *de novo* the whole set of mRNA reads from the total pool of captured cells.

In this study, we aim to test if it is feasible to study the gene expression of a non-model microeukaryote. For this purpose, we use full-length mRNA sequencing to explore and analyse expression profiles of the single-celled chrysophyte *Ochromonas triangulata*. This eukaryotic culture was chosen for its mixotrophic nature where information about feeding behaviour was available, but a genomic reference was not. Cells from two distinct growth phases in culture were sorted using Fluorescence-Activated Cell Sorting (FACS) and single-cell transcriptomic libraries were prepared following the Smart-seq2 protocol, which provides the full-length transcript coverage needed for *de novo* assembly of its transcriptome. This study demonstrates the feasibility to study the gene expression and bacterial interactions of non-model environmental microeukaryotes in a reference-agnostic, culture-free manner, with minimal interference on the samples. Given the abundance and ecological importance of these microeukaryotes in our ecosystems, the study fills a considerable methodological gap that has limited our ability to understand the functional and ecological roles of microeukaryotes in their native habitats.

## Materials and methods

Individual *O. triangulata* cells were sorted from a unialgal non-axenic culture selecting for microeukaryotes with plastids and feeding vacuoles. Transcriptomic libraries for these single cells were made according to Smart-seq2 protocol and then sequenced on NovaSeq 6000 (Illumina). Lacking reference genomes/transcriptomes, *de novo* transcriptomes were assembled by pooling sequences from all cells together and annotated by sequence mapping as well as 3D protein structure mapping. This was followed by downstream analyses, such as differential expression analysis and metabolic mapping. Leaked rRNA in the libraries were used to correctly identify *O. triangulata* as well as the individual microeukaryotes’ associated bacterial communities. Further details of individual steps are mentioned below and in the Supplementary material.

### Culture conditions

Clonal, unialgal, and non-axenic stock cultures of the mixotrophic chrysophyte *O. triangulata* (~8 μm in cell diameter) strain RCC21 (Roscoff Culture Collection) were grown in batches of filter-sterilized K/2 medium ([24], based on artificial seawater prepared according to [25]). The cultures were serially kept at 18°C under a 12 h photoperiod (120 μmol m^−2^ s^−1^ photon flux measured with a QSL-100 spherical sensor, Biospherical Instruments) by diluting 10 times a fraction of each batch into fresh medium approximately every 10 days. No bacterial supplement was added to the culture to sustain growth or promote feeding. Following this routine, representative cells from the fast-growing phase (0.6 doublings per day) were sampled after 2 days from the refreshing date, whereas representatives of the slow-growing phase (0.2 doublings per day) were sampled 11 days after refreshing. *O. triangulata* cell abundance prior to sorting was monitored daily using a CytoFLEX flow cytometer (Beckman Coulter). Cell discrimination was based on both side scatter signal (SSC) and chlorophyll *a* autofluorescence detected in the FL3 channel (EX 488, EM 690/50). Cell abundance of the co-cultured, indigenous bacterial community was based on fixed samples (1% formaldehyde final concentration) kept at 4°C upon fixation and analysed with the Cytoflex within 24 h from sampling after staining with SYBR Green I (Invitrogen) and jointly detected by SSC and FL1 fluorescence (EX 488, EM 525/40).

### Sorting

Cells from both growth phases were stained with LysoSensor Blue DND-167 (Invitrogen), used both as an indicator of cell viability and for presence of food vacuoles (Supplementary Fig. S1). Single-cell sorting was performed with a MoFlo Astrios EQ cell sorter (Beckman Coulter) using 355 and 640 nm lasers for excitation, 100 μm nozzle, sheath pressure of 25 psi and 0.1 μm sterile filtered 1× PBS as sheath fluid. Side scatter was used as trigger channel. Sort decisions were based on gates indicating presence of chlorophyll *a* (640-671/30 vs. SSC) in combination with LysoSensor detection (355-448/59). Singlets were cytometrically selected based on the height-to-area relationship of the pulse (640-671/30 Height-log vs. 640-671/30 Area-log). Individual cells were sorted based on the most stringent single-cell sort settings available (single mode, 0.5 drop envelope) and deposited into 384-well Eppendorf twin.tec PCR plates containing 2 μl of lysis buffer (see Library preparation section for composition). The sample plates were divided into four regions of equal size and cells from either growth phase were distributed in alternating regions to even out technical variation derived from downstream plate processing. The plate holder of the sorter was kept at 4°C. After the sort, the plates were immediately spun down and kept on dry ice until transfer to −80°C for storage. Flow sorting data were interpreted using the sorter-associated software Summit v 6.3.1.

### Library preparation

All plates were processed according to the Smart-seq2 protocol [26] with slight modifications to comply with small-volume handling robots (Smart-seq3, which combines full-length coverage with UMIs, was unavailable to us at the time of this experiment). The oligo-dT (Smart-dTV30, 5′-Biotin-AAGCAGTGGTATCAACGCAGAGTACT30VN-3′) primer, template switching oligo (TSO, 5′-Biotin-AAGCAGTGGTATCAACGCAGAGTACATrGrG+G-3′) primer, and preamplification (IS-PCR, 5′-Biotin-AAGCAGTGGTATCAACGCAGAGT-3′) primer were all modified with a 5′-biotin, which is crucial to increase cDNA yield by avoiding concatenation of TSOs after the first stand reaction [27, 28]. The lysis buffer contained a final concentration of 0.2% Triton-X100 (Sigma), 1 U/μl RNAse inhibitor (cat. no. 2313A, Takara), 2 mM dNTPs (ThermoFisher), Smart-dTV30 primer (IDT), and ERCC RNA Spike-in Mix (cat. no. 4456740, ThermoFisher) diluted 4 × 10^5^ times. The lysis buffer was cold-dispensed in 2 μl fractions using the MANTIS liquid dispenser (Formulatrix). Plates with sorted cells in lysis buffer were thawed and cDNA generation was conducted in 5 μl reactions containing a final concentration of 1× Superscript II buffer, 5 mM DTT, 1 M MgCl_2_, 1 U/μl RNAse inhibitor (cat. no. 2313A, Takara), 5 U/μl SuperScript II Reverse Transcriptase (Invitrogen), and 1 μM TSO (Qiagen). The master mix was dispensed using the MANTIS liquid dispenser followed by mixing for 1 min at 1800 rpm on a plate shaker (Biosan). First strand reaction was run at 42°C for 90 min, followed by 10 cycles of 50°C for 2 min and 42°C for 2 min, with a final 5 min extension at 72°. A 72°C initial denaturation step was omitted as this had shown to have no effect on the results.

Preamplification was performed in 12.5 μl final volume with a final concentration of 1× KAPA HiFi HS RM (Roche) and 10 μM IS-PCR primer. Following 3 min at 98°C, amplification was for 24 cycles of 98°C for 20 s, 67°C for 15 s, and 72°C for 6 min, with a 6 min final extension at 72°C. Bead cleanup of cDNA was automated using the Biomek NXP liquid handler (Beckman Coulter), AMpure beads, and an Alpaqua 384 post magnet with spring cushion technology (Alpaqua). In short, 10 μl beads were added to the 12.5 μl cDNA, the plate was mixed for 1 min at 1800 rpm on a plate shaker and incubated for 8 min before spinning down the liquid and placing the plate on a magnet for 5 min. While on the magnet, the supernatant was removed and 25 μl of freshly prepared 80% EtOH was added and then aspirated after 30 s. The washing was repeated and the plate was left to dry for 2–3 min. Elution of cDNA was done by adding 13 μl of water to the wells and the plate was then mixed before 5 min of incubation at RT. Plates were briefly centrifuged and then put on the magnet for 2 min before transfer of 10 μl to a new plate.

Twenty-two single reactions were randomly chosen across the plate for quantification using Qubit HS DNA ready mix, of which 11–15 samples were also run on the Bioanalyzer using the HS DNA chip (Agilent).

### Sequencing

Nextera XT libraries were prepared in 5 μl reactions where all reagent volumes had been scaled down 10-fold. The purified cDNA was diluted to ~200 pg/μl based on the average cDNA concentration of the 22 randomly chosen reactions. The amplicon tagment mix (ATM) and tagment DNA buffer (TD) reagents were premixed and distributed in a 384-well plate before adding cDNA using the Mosquito HV contact dispenser (SPT Labtech). Barcodes of the Illumina Nextera DNA UD index set A-D (Illumina) were diluted 1:5 prior to the library amplification. Reactions were run for 12 cycles according to cycling conditions recommended by the manufacturer. For all steps, including manual mixing, a plate shaker run at 1800 rpm and a plate spinner were used. Single reactions from each plate were pooled and purified using 1:0.8 AMPure XP beads. The two pools from each plate were run on separate lanes on a NovaSeq 6000 SP v1.5, PE 1× 150 bp, including 1% PhiX spike-in. All sequence data generated in this project have been deposited in the European Nucleotide Archive (ENA) at EMBL-EBI and made publicly available under accession number PRJEB60973.

### *De novo* transcriptome assembly and read mapping

Non-rRNA reads were run through kraken2 v2.1.2 [29] to identify contaminants. Trinity [30] was used for *de novo* combined assembly of the *O. triangulata* partial transcriptome as a reference. Reads from all the individual cells were combined for this task. Following assembly, reads from individual cells were aligned to the reference transcriptome using Bowtie [31] and transcript abundance was estimated using RNA-Seq by Expectation Maximization (RSEM) [32]. The expression was normalized for each cell and all downstream expression values use transcripts per million (TPM) units.

### Clustering and transcript analysis

Before clustering, data from cells with very low read coverage (fewer than 50 000 reads) were removed. Additionally, contigs that were observed in only a single cell or had over 20 million TPM across the population were filtered out to avoid under- and over-representation bias, respectively. The transcript count matrix was dimensionally reduced using *t*-SNE (*t*-distributed stochastic neighbourhood embedding). Further, clustering was performed using the DBSCAN (density-based spatial clustering of applications with noise) algorithm (five minimum points within *ε* = 4.5). Transcript accumulation and transcript coverage curves following rarefaction were generated using the iNEXT R package [33] analogously to procedures used for obtaining curves of species accumulation and species coverage [34], using as counterparts transcript identity for species identity and cell identity for sample site.

### Transcriptome annotation

The *de novo* assembled transcriptome was annotated using blastx as implemented in the BLAST+ suite [35]. UniProtKB/Swiss-Prot was used as the database for the annotation. MGkit [36], AGAT (v1.1.0, [37]), and TransDecoder (v5.5.0, [38]) were used to obtain only the annotated parts of the assembled contigs. The longest isoforms were selected using a custom python script. Reads from the individual cells were re-mapped to the annotated transcriptome for further expression analysis.

### Differential expression analysis

Pairwise differential expression analysis was carried out using DESeq2 [39]. The analysis was performed with reads mapped to both the assembled transcriptome and the annotated transcriptome separately. Transcripts with a Benjamini–Hochberg adjusted *P*-value <.05 were considered as differentially expressed.

### Metabolic mapping

The expression matrix of annotated transcripts was mapped to the Kyoto Encyclopedia of Genes and Genomes (KEGG) pathway database. Pathview [40] was used to map mean expression levels of proteins in the three clusters to metabolic maps or pathways obtained from the KEGG Orthology database. To reduce the number of maps to a tractable number, only maps with at least 1 matched protein, 0.25 match ratio (number of matched proteins/number of proteins in pathway), and 0.1 differential expression ratio (number of differentially expressed proteins/number of matched proteins) were retained. The remaining pathways were manually inspected and pathways where the only differentially expressed genes were not specific to the pathway were removed.

### 3D structure-based transcriptome annotations

70 447 open reading frames, identified using TransDecoder (v5.5.0, [38]), were selected from 150 823 transcripts after filtering out transcripts that were present in a single sample or had a cumulative TPM of over 20 000 000. Of these, very large proteins (>800 aa) were excluded, and 3D structures were generated for 66 973 proteins using ESMFold [41]. After removing structures with low confidence (predicted local distance difference test (pLDDT) ≤ 0.5), 54 721 proteins remained. Read mapping was done, using the same reads, against the corresponding transcript open reading frames (ORFs), which was a subset of the assembled transcriptome, using RSEM [32]. Structural search was performed using Foldseek [42] against the Protein Data Bank (PDB; [43]) as well as the AlphaFold structures of the Uniprot/Swiss-Prot database (AFDB; [44]) with an *e*-value cut-off of 10. Differential expression analysis using DESeq2 [39] and metabolic mapping using pathview [40] was conducted on the reads mapped to these PDB and AFDB annotated proteins. Protein functions, in the form of Enzyme Commission (EC) numbers, and Gene Ontology:Biological Processes (GO:BP) terms were predicted via graph convolutional networks using DeepFRI [45]. Gene set enrichment analysis was performed using fgsea [46] with the predicted GO:BP terms as the gene sets and the list of differentially expressed genes as the preranked list.

### Detection and annotation of rRNA reads

Detection of sequence reads originating from rRNA was carried out with RiboDetector [47]. For taxonomic classification of putative rRNA sequences, reads of 150 nucleotides or longer and represented at least twice in the whole dataset were annotated using the SINTAX algorithm implementation in VSEARCH [48, 49] against the PR2 Reference Sequence Database version 4.14.0 [50] for eukaryote identification and against the SILVA 138 SSU Ref NR 99 [51] for prokaryotic community identification. Prokaryotic data were filtered to remove common human and laboratory-associated contaminants [52]. Alpha and beta diversity of the associated prokaryotes was analysed using the *R* packages *mia* [53] and *vegan* [54]. Differential abundance was determined by a consensus of DESeq2, LinDA [55], MaAsLin2 [56], ANCOM-BC [57], and ALDEx2 with an adjusted *P* < .05 (Benjamini–Hochberg correction) [58].

## Results

### *De novo* transcriptome assembly reveals three distinct metabolic states in unicellular eukaryote

**Figure 1:**
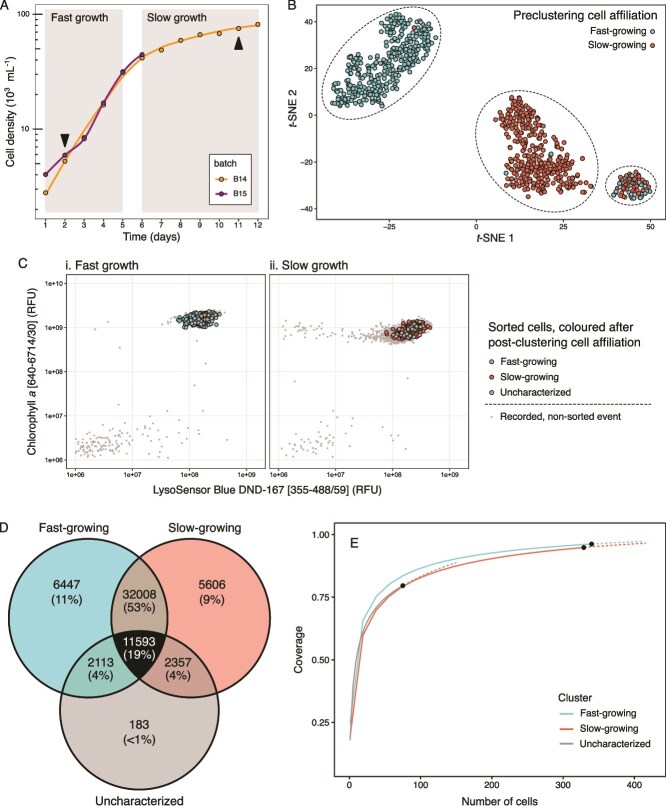
Sampling, sorting, and clustering *Ochromonas triangulata*. (A) Growth curves of source cultures for cells during exponential growth stage (fast growth) and linear growth stage (slow growth). Batch labels refer to the source culture for cells in slow (B14) and fast growth (B15). Arrowheads indicate the sampling points for each stage. (B) *t*-SNE visualization of expression clusters. Each dot represents one cell color-coded based on its sampling origin. Clusters identified by DBSCAN are enclosed by a dotted line. (C) Flow cytograms showing placement of FACS-sorted *O. triangulata* cells (coloured dots) for each growth stage, with cells from the uncharacterized third cluster coloured grey. (D) Venn diagram showing the number of shared and exclusive transcript sets among postclustering groups. (E) Partial transcriptome coverage plot for each postclustering group. The black dots indicate sample size, the solid lines follow sample rarefaction, and the dotted lines represent an extrapolation based on the rarefaction model for each group.

*Ochronomas triangulata* cells sampled from two distinct growth stages (Fig. 1A) were sorted based on the joint signal of chlorophyll *a* and LysoSensor Blue (Fig. 1C and Supplementary Fig. S2). A total of 744 single-cell mRNA libraries were sequenced, yielding a median of 1.4 M reads per cell. *De novo* assembly of reads pooled across all cells produced a transcriptome of ~166 000 contigs (median length 432 bases), which reduced to 60 307 (median length 835 bases) after removing contigs that were not observed in at least two cells (Supplementary Fig. S3). After mapping reads to the assembly, 23 cells that failed to map at least 50 000 reads to the working transcriptome were excluded from further transcriptomic analysis. Of the remaining set of 721 cells, *t*-SNE dimensionality reduction and DBSCAN clustering of expression profiles correctly identified 646 cells with their sorting-based affiliation (Fig. 1B). Unexpectedly, a third, as yet uncharacterized, cluster of cells originating from both the fast (37 cells) and slow (38 cells) sort groups were observed and will henceforth be referred to as the uncharacterized cluster/cells.

In total, 19% of the contigs were present across all three expression clusters and 53% were shared by only the fast- and slow-growing clusters, whereas less than 1% were unique to the uncharacterized cluster (Fig. 1D). However, all expression clusters showed coverage of cluster-specific transcriptomes at or above 80%, with the fast- and slow-growing clusters very close to full transcriptome completeness (Fig. 1E).

### Core metabolic activity occurs in three distinct levels in the three clusters

**Figure 2:**
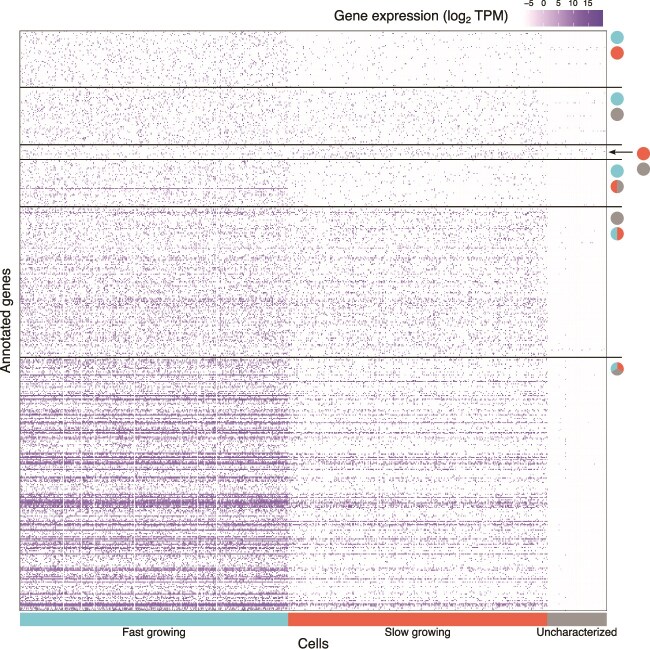
Heatmap of annotated genes that were differentially expressed between different *Ochromonas triangulata* growth stages. Coloured rectangles in the horizontal axis indicate cell affiliation to clustering group. Circle pairs indicate pairwise comparisons between stages. Those cases where gene expression in one group is significantly different to that of the other two groups, the joint is represented by a split circle in which the color of each half encodes the identity of each member. A three coloured circle represents cases in which gene expression differs significantly for any possible pairwise comparison. The colours in circles and rectangles correspond to fast-growing (cyan), slow-growing (red), and uncharacterized (grey) stages.

**Figure 3:**
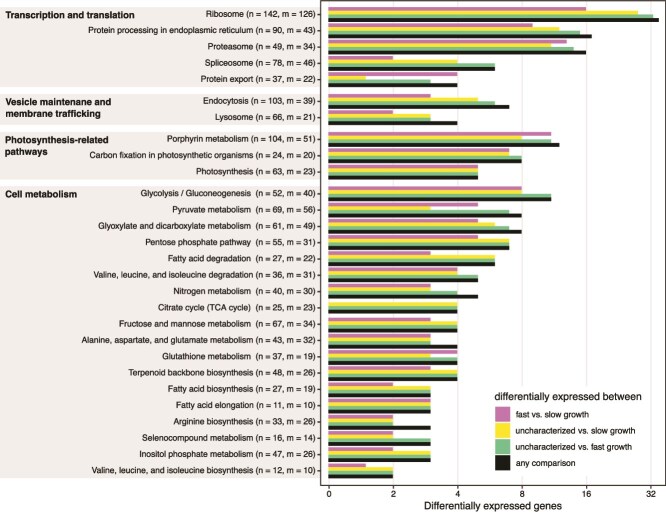
Summary of KEGG pathways associated to multiple differentially expressed genes. For each pathway label, *n* indicates the total number of genes that belong to the pathway and *m* the number of genes for which expression was recovered in our transcriptomes.

Only 17 883 transcripts out of the 60 307 contigs could be annotated using sequence homology against the UniProt database (see Supplementary Fig. S4 for the proportions of annotated transcripts per expression cluster), corroborating the underrepresentation of annotated protist sequences in public databases [59]. Pairwise comparisons among the three distinct cell clusters revealed 538 differentially expressed annotated genes (DEGs; Fig. 2). Mapping these DEGs against KEGG Orthology pathways identified four main pathway families: transcription and translation processes, vesicle maintenance and membrane trafficking, photosynthetic activity, and carbohydrate metabolism (Fig. 3). DEGs in the KEGG pathways detected from any pairwise comparison against the uncharacterized group were in all instances and without exception, downregulated in the latter.

Three pathways related to transcription and translation (ribosome, protein processing, and proteasome) featured the highest number of differentially expressed genes. In addition, two other key components in the progression from transcript to functional protein (spliceosome, protein export) also had a small number of differentially expressed genes. Downregulation of genes involved in such processes was prevalent in the uncharacterized cells (28 and 33 genes when comparing it to the slow-growing or fast-growing cells, respectively). This agrees with the expression level pattern across the groups (Fig. 2) and suggests reduced transcriptional activity in the uncharacterized group when compared to the other two.

The second pathway family (endocytosis and lysosome pathways) provides insights into endocytic behaviour, revealing joint downregulation of clathrin and AP-2 (both essential coating components of vesicles resulting from endocytosis) in the uncharacterized group as well as high expression of lysosomal proteases (homologues of tripeptidyl-peptidase 1 and cathepsin A, B, D, F, and X) in fast- and slow-growing expression clusters in parallel with negligible expression of these proteases in the uncharacterized group. These observations, although limited, provide evidence of active lysosomal digestion in the growing cells, which in contrast seems to be absent from the uncharacterized cells.

**Figure 4:**
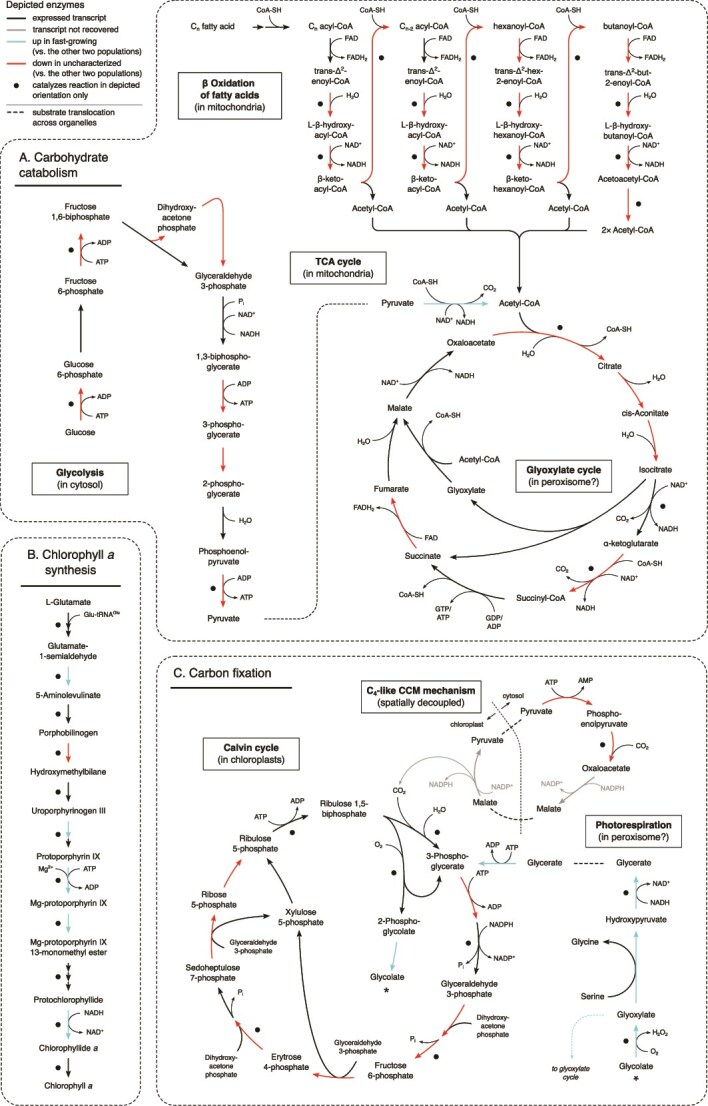
Differential expression in key metabolic pathways involving (A) carbohydrate catabolism (glycolysis, tricarboxylic acid cycle, and β oxidation of fatty acids), (B) chlorophyll *a* synthesis, and (C) carbon fixation (Calvin cycle, CCM mechanism, and photorespiration). Coloured arrows represent enzymes that catalyse the depicted reactions. Black dots indicate enzymes that only catalyse the reactions in the depicted direction. Dashed lines indicate substrate translocation across organelle boundaries.

The third and fourth pathway families, represented by the pathways associated to photosynthesis and carbohydrate metabolism, harbour the highest number of DEGs featured in the whole set of KEGG and provide the clearest picture associated to physiological differences between cell stages (Fig. 4). In all cases, DEGs in this category showed high expression in the fast-growing cells, and low in the uncharacterized cells, whereas expression was either intermediate or comparable to the fast-growing in the slow-growing cells.

Widespread downregulation of carbohydrate catabolism is a pronounced feature of the uncharacterized cells, apparent through the downregulation of key enzymes of the glycolysis pathway (phosphofructokinase-1 and pyruvate kinase), the β oxidation of fatty acids (enoyl-Coenzyme A (CoA) hydratase, β-hydroxyacyl-CoA dehydrogenase, and acyl-CoA acetyltransferase), and the tricarboxylic acid (TCA) cycle (citrate synthase) (Fig. 4A). Expression of all enzymes involved in β oxidation and most enzymes in the TCA cycle was recovered in all three clusters. Low expression of genes mediating such pathways in the uncharacterized cluster implies low levels of energy production for cell maintenance when compared to the other two groups.

In contrast, observable in the fourth KEGG family pathway with the highest number of DEGs (porphyrin metabolism; Fig. 3), exacerbation of chlorophyll *a* synthesis manifested in the fast-growing stage (Fig. 4B). It involves the upregulation of five enzymes in the pathway, including magnesium chelatase, which catalyses the first committed step of chlorophyll *a* production, and protochlorophyllide reductase, which generates chlorophyllide *a*, the immediate precursor of chlorophyll *a*. A putative increased chlorophyll *a* production in the fast-growing stage is in accordance with an enhanced chlorophyll fluorescence signal in the sorting cytograms (Fig. 1B).

Downregulation of key enzymes of the Calvin cycle in the uncharacterized cells (fructose 1,6-biphosphatase and fructose-bisphosphate aldolase, both catalysing unidirectional reactions, Fig. 4C) indicates little or no carbon fixation in this group. At the same time, upregulation of photorespiration enzymes in the fast-growing stage suggests high CO_2_ fixation activity associated to these cells, because this is a compensatory mechanism to counteract the efficiency loss in carbon fixation due to the oxygenase activity of rubisco [60]. Photorespiration is expected to occur in peroxisomes [61], which are not characterized in chrysophytes but are reported in other stramenopiles based on microscopic and genomic evidence [62, 63]. This, in combination with increased chlorophyll *a* production, indicates fast growth conditions are facilitated by photosynthesis.

Among DEGs not associated to a relevant KEGG pathway, only three show significant upregulation in the uncharacterized cluster. Their UniProt-based annotation corresponds to prokaryotic entries in the database, although the three genes have also eukaryotic homologues. Additionally, many genes unique to this uncharacterized group (42 of the 183 contigs) all had prokaryotic hits as basal annotation, 22 of which lack any eukaryotic homologue.

### Structural homology analysis pipeline adds annotation and upholds sequence homology-based inferences

**Figure 5:**
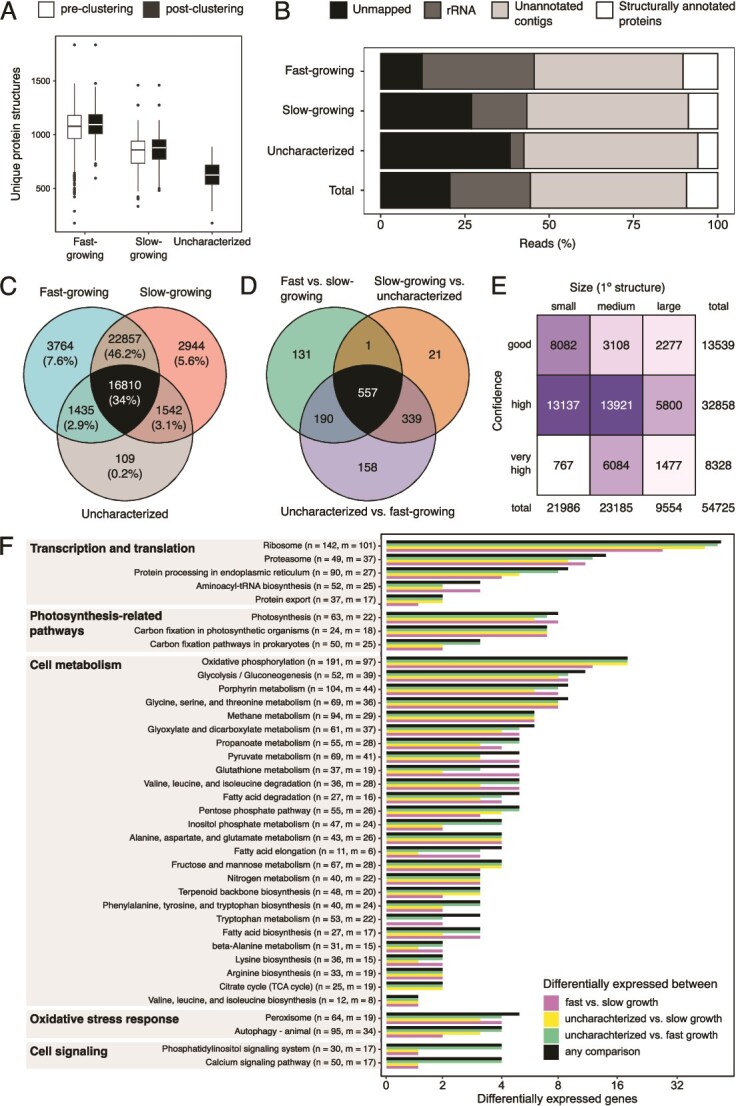
Protein tertiary structure-based analyses. (A) Distributions of protein richness per cell. (B) Relative proportion of total reads that either did not map to the assembled transcriptome, were assigned as ribosomal, or mapped to unannotated contigs or structurally annotated proteins (C) Venn diagram showing the number of shared and exclusive protein sets among postclustering groups. (D) Venn diagram showing the number of shared and exclusive protein sets that were differentially expressed among the postclustering groups. (E) Distribution of the predicted protein structures on protein sequence length (no. of amino acids: small (0, 200], medium (200, 400], large (400, 800]), and structure confidence (pLDDT scores: good (0.5, 0.7], high (0.7, 0.9], very high (0.9, 1]) axes. (F) Summary of KEGG pathways associated to multiple differentially expressed genes (*n* = no. of proteins in pathway, *m* = no. of those proteins recovered). Functions and pathways also identified by sequence homology (Fig. 3) include transcription and translation (ribosome, protein processing in endoplasmic reticulum, and proteasome), photosynthesis (porphyrin metabolism, photosynthesis, and carbon fixation in photosynthetic organisms), and cell metabolism (glycolysis, pyruvate metabolism, citrate cycle, fatty acid biosynthesis, and elongation) associated pathway groups.

ESMFold successfully predicted the 3D structures for 54 725 proteins (Fig. 5E and Supplementary Tables S1 and S2), including 32 858 proteins with high confidence (0.7 < pLDDT ≤ 0.9) and 8328 proteins with very high confidence (pLDDT > 0.9). Cells in fast-growing, slow-growing, and uncharacterized clusters featured a median of 1094, 878, and 624 expressed protein-coding genes, respectively (Fig. 5A). Over 80% of the predicted structures were common to both fast- and slow-growing clusters, whereas only 0.22% were unique to the uncharacterized cluster (Fig. 5C). The distribution of predicted protein encoding genes expressed in the three clusters was similar to the distribution based on sequence homology. A median of 164 184, 122 519, and 75 196 RNA reads mapped to the fast-growing, slow-growing, and uncharacterized cells, respectively, based on the transcripts associated to predicted structures. Overall, these comprised 9.31% of all reads sequenced, which is similar to the 10.5% reads mapping to the transcripts annotated by sequence similarity analyses (Fig. 5B and Supplementary Fig. S5).

Unlike sequence similarity-based annotation, where only 29.6% of the transcripts were successfully annotated, over 99.9% of the transcripts were successfully annotated based on structural homology. A total of 1397 of these transcripts were differentially expressed between the comparisons, with significant differences in all three comparisons for 557 transcripts bearing high expression in fast-growing cells, intermediate expression in slow-growing cells, and low expression in uncharacterized cells (Fig. 5D and Supplementary Fig. S6). Additionally, there were 339 transcripts that had comparable expression between fast- and slow-growing cells, but having low expression in uncharacterized cells.

Between sequence-similarity–based analysis and structure-similarity–based analysis, there was large overlap among the KEGG pathways associated with differentially expressed genes (Figs 2 and 5F); these pathways represented transcription and translation, photosynthesis, and cell metabolism associated pathway groups. Additionally, pathways associated with cell cycle (focal adhesion and oocyte meiosis) and oxidative stress (autophagy and peroxisome) were identified. This was further evident from gene set enrichment where a total of 100, 91, and 69 GO:BP gene sets were significantly enriched for in the three comparisons: fast- vs. slow-growing, uncharacterized vs. fast-growing, and uncharacterized vs. slow-growing comparisons. These enriched gene sets included GO terms for cellular response to oxidative stress (GO:0034599), oxygen radical (GO:0071450), reactive oxygen species (GO:0034614), stress (GO:0033554), as well as DNA repair (GO:0006281). Some pathways’ enrichment suggested bacterial symbiosis, for instance, interspecies interaction (GO:0044419) and symbiotic interaction (GO:0044403) and potentially bacterial invasion, for instance, interaction with host (GO:0051701), entry into host (GO:0044409), and movement in host (GO:0052126, Supplementary Tables S3–S5).

### rRNA read carry-over enables identification of microeukaryote and distinct associated prokaryotes

Overall, 241.7 M reads were predicted to be of rRNA origin across all samples (24% of the total), although rRNA read coverage was uneven across cell types, constituting 33%, 16%, and 4% of the fast-growing, slow-growing, and uncharacterized cells, respectively (Supplementary Fig. S5). After discarding singletons and reads shorter than 150 nucleotides, the resulting set was successfully de-replicated into 4.5 million unique sequences. Of these, one third (33%) contained useful information for taxonomic annotation and could be classified with confidence (>0.8 bootstrap support). Annotated read recovery across samples was sufficient (median 106 688 sequences per sample) to correctly annotate most cells (88%) as *O. triangulata* (median 23 811 per sample). The remaining 75 cells belonged, without exception, to the uncharacterized cluster.

Median rRNA abundance in the uncharacterized cluster, across all rRNA genes, was low (~43 000 reads per cell). A broadly reduced expression landscape, which also includes lower demand for rRNA (as suggested by generalized downregulation of ribosomal proteins, Fig. 3), might explain insufficient rRNA coverage for unambiguous annotation of cells characterized by low transcriptional activity.

The 16S rRNA read recovery across the samples was relatively low (median 2807 reads per sample) but saturated (Supplementary Fig. S7), and sufficient to characterize the prokaryotic community composition associated with individual *O. triangulata* cells.

**Figure 6:**
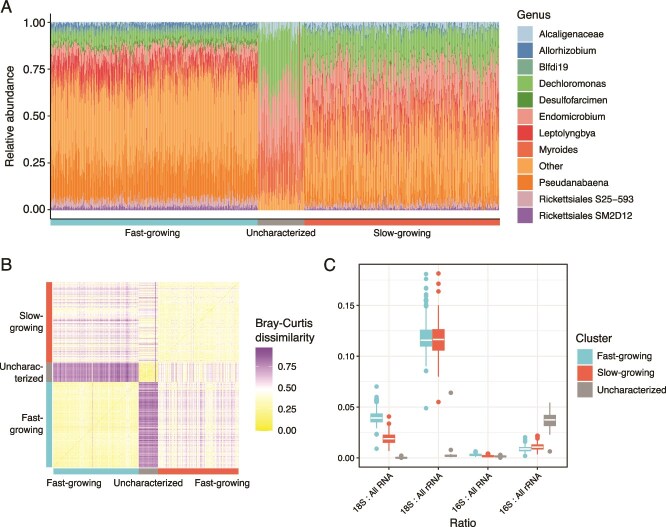
The prokaryota associated with the *O. triangulata* cultures. (A) Relative abundance of the 11 most abundant prokaryotic genera associated with the different expression clusters. (B) Bray–Curtis dissimilarity of each sample’s associated prokaryota with the rest of the samples. (C) Ratios of 18S and 16S rRNA to the rest of the RNA or rRNA obtained from the samples.

The prokaryotic communities of the uncharacterized cells were more internally uniform (Fig. 6A and B; median Bray–Curtis dissimilarities 0.23 within the group) compared to the fast-growing and slow-growing cells (median Bray–Curtis dissimilarity 0.31 and 0.38 within the groups, respectively; Fig. 6B). The prokaryotic communities were significantly different between the fast- and slow-growing (Permuttional Analysis of Variance (PERMANOVA): *R*^2^ = 0.32, *P* = .001; median Bray–Curtis dissimilarity 0.50), fast-growing and uncharacterized (PERMANOVA: *R*^2^ = 0.60, *P* = .001; median Bray–Curtis dissimilarity 0.80), and slow-growing and uncharacterized cells (PERMANOVA: *R*^2^ = 0.22, *P* = .001; median Bray–Curtis dissimilarity 0.47). Bacterial alpha diversity (richness) also was significantly different between the three groups (Supplementary Fig. S7C).

The proportion of 16S rRNA to total rRNA was significantly higher in the uncharacterized cell cluster compared to the fast- and slow-growing groups (Wilcoxon *P* < 2.2 × 10^−16^ and *P* < .009, respectively), whereas the proportion of 16S rRNA to total RNA was not significantly different between any of the groups (Fig. 6C). This is in contrast with the proportion of the 18S rRNA to all rRNA and all RNA reads, where the 18S rRNA proportion for the uncharacterized group was significantly lower than the others (Wilcoxon test *P* < 2.2×10^−16^ and *P* < 2.2×10^−16^, respectively). The differentially abundant families (Supplementary Fig. S7C and Supplementary Table S6) between the cell clusters include *Flavobacteriaceae*, *Endomicrobiaceae*, *Devosiaceae*, *Alcaligenaceae*, *Rhodocyclaceae, Xanthomonadaceae* (lowest abundance in the fast-growing population, highest abundance in the uncharacterized population), and *Comamonadaceae*, *Holosporaceae*, *Prolixibacteraceae*, *Bdellovibrionaceae*, and *Cyanobacteriaceae* (lowest abundance in the uncharacterized population, highest abundance in the fast-growing population).

## Discussion

Single-cell transcriptomic profiling of the microbial eukaryote *O. triangulata* was technically feasible with minimal sample processing and without the need of a reference genome. Although resources like the Marine Microbial Eukaryotic Transcriptome Sequencing Project [59], containing 678 functionally annotated transcriptomes of a number of marine microbial eukaryotes (now available as a database of predicted protein sequences [64]) are currently available, their taxonomic breadth is relatively limited. However, given the high diversity of microeukaryotes in most environments, it cannot be expected that randomly sampled microeukaryotes would have reference genomes available. In our case, no reference for *O. triangulata* was available in the database, and assembly against the reference from other *Ochromonas* species yielded very low mapping. In contrast, the SmartSeq2 protocol, used to generate the mRNA libraries of FACS-sorted single cells of *O. triangulata*, provided sufficient output while preserving the full-length transcript information necessary to generate a working transcriptome assembled *ad hoc*. Using this transcriptome as a basis for the analysis of gene expression, we were able to discern three distinct cell clusters of *O. triangulata* originating from two contrasting growth stages, therefore uncovering a third uncharacterized group of cells that was not expected *a priori*.

Transcript recovery for each of these three populations was satisfactory. Concerns about the need of very large sample sizes to discern expression patterns in microbial eukaryotes have been raised previously [20], indicating that sampling sizes well above 100 individuals would be necessary. Such concerns are grounded on the joint effect of the low numbers of mRNA molecules coexisting in a small microbial eukaryote [20] together with the widespread occurrence of gene transcription in successive bursts [65]. Indeed, our results show that transcriptome recovery at cell sampling depth (represented by black dots in Fig. 1E) is far from saturation in all three groups. Nonetheless, at least for *O. triangulata*, 100 cells would have been sufficient to recover ~80% of the transcriptome in each of the three groups (Fig. 3E). We therefore suggest that sampling in the order of 100 individuals might be enough to characterize microeukaryote populations based on single-cell expression profiles. As a control, a bulk RNA transcriptome or metatranscriptome could be generated too.

Although we found no technical limitations in deploying scRNA-seq, annotation issues are still pervasive. In our dataset, two-thirds of the comprehensive transcriptome remained unannotated (Supplementary Fig. S4). Consequently, of all the data generated in our experiment, only ~11% of the reads contributed to our understanding of mRNA expression that could be associated with putative biological functions (Supplementary Fig. S5). In contrast, the dataset is characterized by a large proportion of reads that mapped to transcripts devoid of known function (45% in our dataset) and therefore much biological significance remains unavailable due to annotation gaps. Unavoidably, organisms that are poorly represented in annotation databases (as is the case for most microeukaryotes) will be subject to such constraints. Experimental studies targeting specific genes and sequencing whole genomes for environmental microeukaryotes will aid in mitigating this problem.

We explored predictive structural annotation to mitigate this phenomenon. We obtained tertiary structures for ~55 000 proteins and successfully annotated over 99% of them. Read mapping against these proteins’ transcripts however remained on par, at ~10%, with mapping against ~20 000 sequence-annotated transcripts. Such low mapping to transcripts of known function after structural analysis could indicate that many contigs and transcripts from the *de novo* assembly may not encode proteins at all.

The problems associated to transcripts with unknown function are well exemplified by the uncharacterized cell cluster. This cell group had fewer well supported unique transcripts that accounted for a good representation of the expression landscape of the group (covering ~80% of its full transcriptome). In turn, most of these transcripts were shared with one or both other two groups, whereas only 1% were unique to this group. The question of what this seemingly uncharacterized cluster represents remain enigmatic. We speculate that these cells might be under colonization by a bacterial community, possibly involving pathogens or parasites, as exemplified by presence of members of *Endomicrobiaceae* family which are known to endosymbionts of protozoa [66]. This scenario would be consistent with the prokaryotic nature of at least a portion of the mRNA transcripts unique to the uncharacterized group. This possibility would be compatible with the very erratic recovery associated with these transcripts, since the recovery of non-polyadenylated prokaryotic mRNA, likely caused by random mRNA capture in a similar mechanism driving rRNA leakage, would be expectedly inconsistent. In addition, the relatively high proportion of transcripts that are shared between the uncharacterized cluster and either of the other two groups (slightly above 2000 transcripts in both cases; Fig. 3C) agrees with the hypothesis that the uncharacterized cluster is the result of bacteria invading cells that were originally from the other two groups.

Despite the presence of transcripts with unknown function, differential expression of genes involved in quasi-universal cellular pathways, and therefore well represented in annotation databases, provided insight into the functional state of *O. triangulata* beyond the uncharacterized group. For example, enhanced photosynthetic activity in combination with seemingly increased CO_2_ fixation were characteristic of cells in fast exponential growth. This, together with a concurrent peak in bacterial abundance during this phase seemed to indicate that a boost in *O. triangulata* growth, could be enabled by its heterotrophic activity (Supplementary Fig. S8). When prey declined into a stable, potentially predation-resistant community, *O. triangulata* growth transitioned into a slower growth rate driven mostly by photosynthesis. This would be in line with what has been shown before for *Ochromonas* isolate CCMP1393 [67, 68] and would delineate *O. triangulata* as a mainly phototrophic constitutive mixotroph that could benefit from prey uptake to accelerate growth (group C mixotroph *sensu* Jones [69]).

Results from the structure-based analysis corroborated those from the sequence-based analysis in terms of read mapping, transcript diversity, and differential expression between clusters, as well as metabolic mapping. Additionally, improved structure and structure–function annotation, based on predicted protein structures, as compared to sequence-based annotation, revealed enrichment in pathways associated with oxidative stress as well as interspecies interaction in the uncharacterized expression cluster, providing further evidence that this uncharacterized population could be composed of cells that are close to dormancy or have been invaded by bacteria. Beyond protein expression, another additional source of useful information that was featured in our dataset came from rRNA annotation. Although Smart-Seq2 targets specifically mRNA, the cellular amount of rRNA in a eukaryotic cell is vast enough to allow considerable leakage of rRNA reads into the dataset. This phenomenon, typically regarded as a nuisance, can be used to the researcher’s advantage when taxonomic affiliation of a cell is unknown, as would be the case from a natural sample. In our dataset, about one-quarter of the total reads corresponded to rRNA, although it was not equally distributed across cell clusters. This can become limiting when read coverage per cell is relatively low, since we needed >100 000 reads to correctly annotate *O. triangulata* cells based on direct annotation of rRNA reads. The reason for this might be that the taxonomical information conveyed by these sequences is generally poor because they can originate anywhere in the rRNA operon, much of which is phylogenetically ambiguous. In the case of the uncharacterized group, where only 4% of the reads overall originated from rRNA, annotation becomes misleading. The potential prokaryotic nature of many reads associated to the uncharacterized cell group could also explain the difficulty in proper taxonomic annotation for members of this group. Nevertheless, when read support is high, cells could be taxonomically annotated with confidence.

The carry-over of 16S rRNA reads in the sequencing data permits profiling the bacterial communities associated with individual *O. triangulata* cells. The community compositions were consistent within the clusters but variable between them despite the similarity of the co-cultured bacterial communities (Fig. 6B). The internally most consistent bacterial communities were amidst the uncharacterized cell group whereas more variability was observed within the fast-growing and slow-growing groups. Additionally, the bacterial community composition of the members of the uncharacterized expression cluster did not depend on their origin in the fast- or slow-growing cells. Also, the slight bimodality in the alpha diversity of the uncharacterized cells had no connection to the growth stage.

We speculate that the differences in the bacterial communities between the groups could be explained by grazing behaviour of the *O. triangulata* cells. Initially, low abundance of *O. triangulata* cells would allow the bacteria to grow fast under little grazing pressure. With time and consequently increasing numbers of *O. triangulata*, we speculate that the peak density of the bacterial community is consumed and the community transitions to a grazing-resistant steady state (Supplementary Fig. S8) forcing *O. triangulata* to progress from a fast-growing to slow-growing state. The increased grazing pressure due to higher numbers of *O. triangulata* could selectively affect the community structure of the co-cultured bacteria, which in turn could result in lower stability as well as a shift from the bacterial community associated with the fast-growing population. Unfortunately, the current study lacks community profiling of the co-cultured bacterial cells and therefore we are not able to confirm the similarity of the individual *O. triangulata* bacterial communities to the bulk bacterial community structure of the fast- and slow-growing culture stages.

The specific bacterial community composition within the uncharacterized cell cluster is consistent with the scenario described above, in which bacteria might be colonizing compromised *O. triangulata* cells. This is supported, in addition to the lower abundance of total RNA reads associated to this cluster, by its elevated cell-associated bacterial load, observed as a significantly higher proportion of prokaryotic rRNA in cells from this cluster compared to the other two groups. As the cells in the uncharacterized cluster do not seem to be actively feeding according to the downregulation of key lysosome-related enzymes, colonization by invasive bacteria seems a plausible explanation of the higher prokaryotic rRNA. Finally, the consistent bacterial community composition associated to these cells could be explained by very specific bacterial community members taking part in the invasion. However, this third uncharacterized state was not expected by the authors *a priori*, and the bacterial invasion is one potential explanation. Further study is needed in the future with methods like fluorescence in situ hybridization (FISH), scanning electron microscipy (SEM), and transmission elecctron microscopy (TEM) to provide conclusive evidence.

There are myriad small microeukaryotes that are difficult to culture in laboratory conditions and consequently to study. Our results show that scRNA-seq is technically apt to deal with such small microeukaryotes without sample manipulation, even those lacking a reference genome. Although potential for detailed mechanistic understanding of functional states is limited for organisms that are poorly represented in public databases, such as microeukaryotes, potential for discovery of unexpected and diverse expression consortia remains a key asset of scRNA-seq. Taken together, the combination of 18S rRNA annotation with expression-driven cell clustering and structural homology-driven protein annotation holds promise as a powerful tool to characterize community structure and function from natural samples beyond 18S rRNA gene amplicon sequencing.

## Supplementary Material

Supplement

Supplement_Tables_wraf046

## Data Availability

All sequence data generated in this project have been deposited in the European Nucleotide Archive (ENA) at EMBL-EBI and made publicly available under accession number PRJEB60973. They are available at the following https://www.ebi.ac.uk/ena/browser/view/PRJEB60973.
