# Supplementary material for: Cellular heterogeneity in metabolism and associated microbiome of a non-model phytoflagellate
Source: ISME J. 2025 Mar 9;19(1):wraf046. doi: 10.1093/ismejo/wraf046 (PMC11973420; doi:10.1093/ismejo/wraf046)
Supplement: Supplement [file supplement.pdf]

Supplementary information

Supplementary methods

Pre-processing reads

Raw reads were first processed using Trim Galore v0.6.10 and Cutadapt v1.18 to remove the Nextera Transposase adapter sequence (CTGTCTCTTATA). Additionally, a Phred quality score cutoff of 20 was enforced. The minimum required sequence length for both reads before a sequence pair gets removed was set to 20 bp, with a maximum trimming error rate of 0.1.

Ribosomal RNA reads were separated from the rest using Ribodetector v0.2.7 ensuring that "only high confident rRNAs are classified as rRNA and the rest output as non-rRNAs".

Non-rRNAs were put through kraken2 v2.1.2 to identify contaminants. The reference database consisted of NCBI taxonomic information, as well as the complete genomes in RefSeq for the bacterial, archaeal, and viral domains, along with the human genome, and a collection of known vectors (UniVec_Core). A confidence score threshold of 0.60 was used, in accordance with guidance from Wright R.J. et al. (https://doi.org/10.1099/mgen.0.000949).

De novo transcriptome assembly and read mapping

Trinity v2.14.0 [35] was used for *de novo* combined assembly of the *O. triangulata* partial transcriptome as a reference from the non-rRNA reads. Reads from all the individual cells were combined for this task.

Following assembly, reads from individual cells were aligned to the reference transcriptome using Bowtie v1.2.3 [36] and transcript abundance was estimated using RSEM v1.3.3 [37]. The alignment and transcript abundance estimation was performed using a perl script from the writers of Trinity called align_and_estimate_abundance.pl with the following parameters: --all --best --strata -m 300 --chunkmbs 512.

The expression was normalized for each cell and all downstream expression values use transcripts per million (TPM) units.

Clustering and transcript analysis

Before clustering, data from cells with very low read coverage (fewer than 50 000 reads) were removed. Additionally, contigs that were observed in only a single cell or had over 20 million TPM across the population were filtered out to avoid under- and over-representation bias, respectively. The transcript count matrix was dimensionally reduced using *t*-SNE (*t*-distributed Stochastic Neighbourhood Embedding) using the Rtsne R package with 0.0 theta, partial PCA, and random seed set to 123. Further, clustering was performed using the DBSCAN (Density-Based Spatial Clustering of Applications with Noise)

algorithm (5 minimum points within ε = 4.5). The parameters for DBSCAN were determined by drawing the k-nearest neighbours distribution plot. Transcript accumulation and transcript coverage curves following rarefaction were generated using the iNEXT R package [38] analogously to procedures used for obtaining curves of species accumulation and species coverage [39], using as counterparts transcript identity for species identity and cell identity for sample site.

Transcriptome annotation

The *de novo* assembled transcriptome was annotated using blastx as implemented in the BLAST+ suite [40]. UniProtKB/Swiss-Prot was used as the database for the annotation. MGkit [41], AGAT (v1.1.0, [42]) and TransDecoder (v5.5.0, [43]) were used to obtain only the annotated parts of the assembled contigs. TransDecoder LongOrfs was used to filter out predicted proteins smaller than 100 amino acids and TransDecoder Predict to retain the ORFs corresponding to the BLAST hits, while using the default universal genetic code. MGkit blast2gff function was used to convert the BLAST output to gff format and agat_convert_sp_gff2gtf.pl was used to convert the gff output to gtf format. The longest isoforms were selected using a custom python script. Reads from the individual cells were re-mapped to the annotated transcriptome (using Bowtie and RSEM) for further expression analysis.

Differential expression analysis

Pairwise differential expression analysis was carried out using DESeq2 [44] with default parameters. The analysis was performed with reads mapped to both the assembled transcriptome and annotated transcriptome separately. Transcripts with a Benjamini-Hochberg adjusted p-value less than 0.05 were considered as differentially expressed.

Metabolic mapping

The expression matrix of annotated transcripts was mapped to the Kyoto Encyclopedia of Genes and Genomes (KEGG) Pathway database. A total of 258 KEGG metabolic maps were considered (Refer: https://www.genome.jp/brite/ko00001). Pathview [45] R package was used to map mean expression levels of proteins in the three clusters to metabolic maps or pathways obtained from the KEGG Orthology database. To reduce the number of maps to a tractable number, only maps with at least 1 matched protein, 0.25 match ratio (number of matched proteins/ number of proteins in pathway), and 0.1 differential expression ratio (number of differentially expressed proteins/ number of matched proteins) were retained. The remaining pathways were manually inspected and pathways where the only differentially expressed genes were not specific to the pathway were removed.

3D structure-based transcriptome annotations

70447 open reading frames, identified using TransDecoder (v5.5.0, [43]), were selected from 150823 transcripts after filtering out transcripts that were

present in a single sample or had a cumulative TPM of over 20000000. TransDecoder LongOrfs was used to filter out predicted proteins smaller than 100 amino acids and TransDecoder Predict to retain the ORFs corresponding to the BLAST hits, while using the default universal genetic code. Of 70447 ORFs identified, very large proteins (>800 aa) were excluded, and 3D structures were generated for 66 973 proteins using ESMFold [46], partially using the POST API available at https://api.esmatlas.com/foldSequence/v1/ and partially offline using the command line interface tool esm-fold available at https://github.com/facebookresearch/esm. After removing structures with low confidence (pLDDT (predicted local distance difference test) <= 0.5), 54 721 proteins remained. Read mapping was done, using the same reads, against the corresponding transcript ORFs, which was a subset of the assembled transcriptome, using RSEM [37]. Structural search was performed using Foldseek using default parameters [47] against the Protein Data Bank (PDB, [48]) as well as the AlphaFold structures of the Uniprot/Swiss-Prot database (AFDB, [49]) with an e-value cut-off of 10. Differential expression analysis using DESeq2 [44] and metabolic mapping using pathview [45] was conducted on the reads mapped to these PDB and AFDB annotated proteins. Protein functions, in the form of Enzyme Commission (EC) numbers and Gene Ontology: Biological Processes (GO:BP) terms, were predicted using a graph convolutional network based tool called DeepFRI using default parameters [50]. Gene set enrichment analysis was performed using fgsea [51], with number of permutations for preliminary estimation of p-values, with the predicted GO:BP terms as the genesets and the list of differentially expressed genes as the pre-ranked list.

Detection and annotation of rRNA reads

Detection of sequence reads originating from rRNA was carried out with RiboDetector [52] v0.2.7 ensuring that “only high confident rRNAs are classified as rRNA and the rest output as non-rRNAs”. For taxonomic classification of putative rRNA sequences, reads of 150 nucleotides or longer and represented at least twice in the whole dataset were annotated using the SINTAX algorithm implementation in VSEARCH [53, 54] against the PR2 Reference Sequence Database version 4.14.0 [55] for eukaryote identification and against the SILVA 138 SSU Ref NR 99 [56] for prokaryotic community identification. Data was filtered of common human-associated and laboratory contaminants. Alpha and beta diversity of the associated prokaryotes was analysed using the *R* packages *mia* and *vegan*. Differential abundance was determined by a consensus of DESeq2, LinDA, Maaslin2, ANCOMBC, and ALDEx2 with an adjusted $P < 0.05$ (Benjamini-Hochberg correction), all run with default parameters.

Supplementary figures

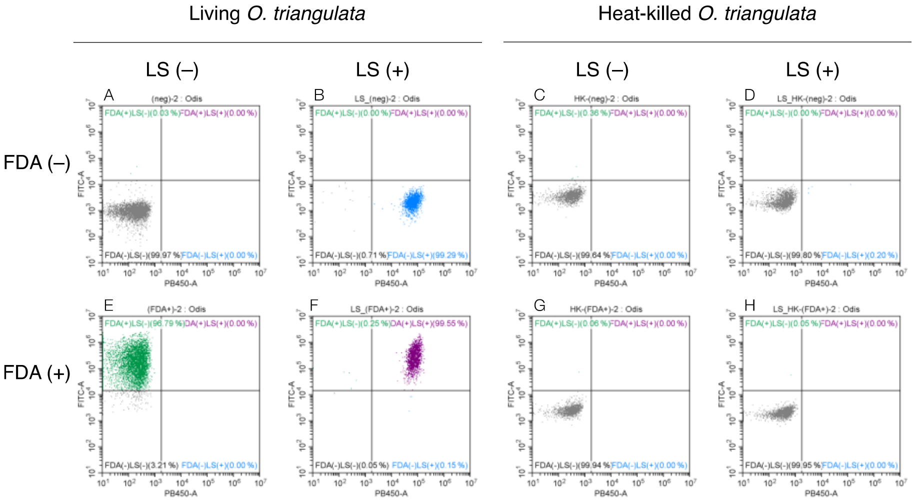

Supplementary Figure 1. LysoSensor Blue (LS) staining as a proxy for cell viability in Ochromonas triangulata. Double staining with LS and fluorescein diacetate (FDA), a commonly used reporter for cell viability (MacIntyre and Cullen 2016) shows that both LS and FDA reveal identical populations of living cells. Cells were heat-killed by immersing cultures in a water bath at 55°C during 15 min, then tempered at 18°C during 30 min prior to staining. Stain final concentrations were FDA 1 µg/mL, LS 0.5 mM. (+) Stain added, (–) stain absent. FDA solutions were freshly prepared and used within 30 min from preparation. All scatterplots show blue fluorescence intensity (EX 405, EM 450/45) on the horizontal axis and green fluorescence intensity (EX 488, EM 525/40) on the vertical axis.

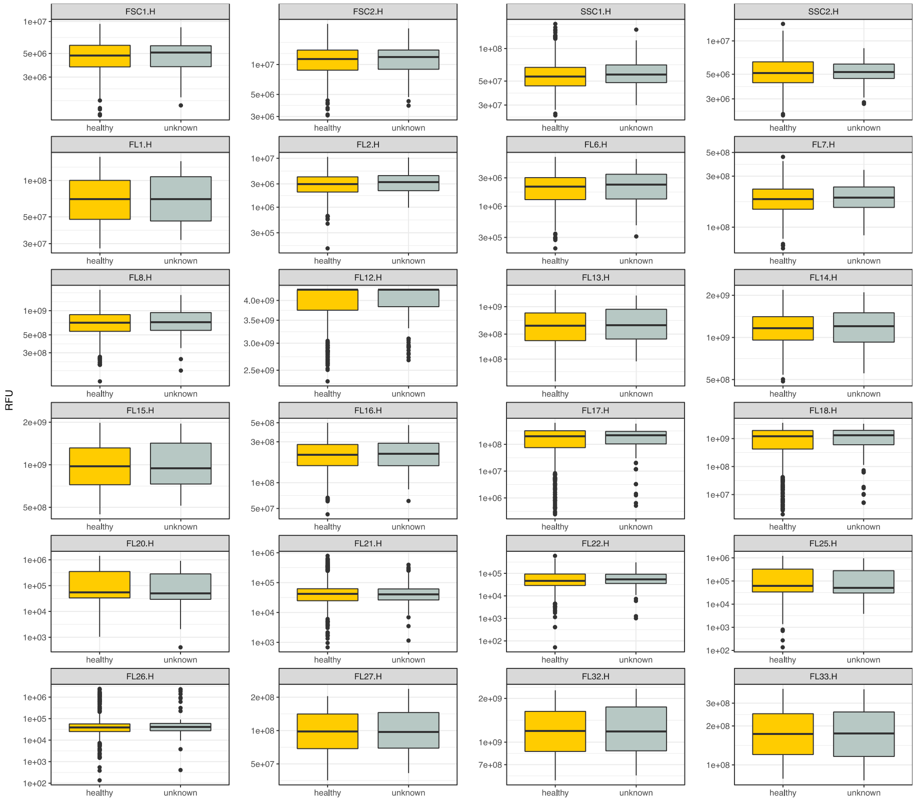

Supplementary Figure 2.. Boxplots showing the distribution of relative fluorescence intensities for all the sorted cells in the fast and slow growth stages (yellow boxes) and in the unexpected cluster (gray boxes). LysoSensor Blue signal was recorded via the FL7 channel, while chlorophyll signal corresponds to the FL32 channel. No significant differences (Wilcoxon signed rank test) are found among these two groups for any of the channels recorded.

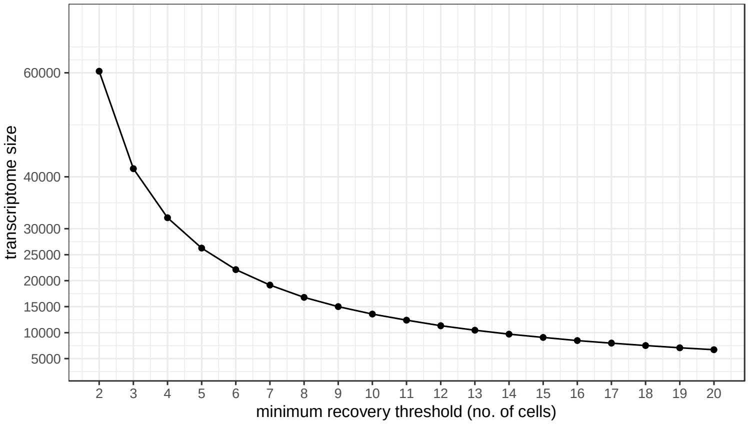

Supplementary Figure 3. Resulting transcriptome size (number of unique transcripts) after successive minimum recovery thresholds (MRTs). The MRT represents the minimum number of cells required to feature a given transcript in order to legitimate the presence of the transcript in the transcriptome. In our analysis, only transcripts recovered at MRT of 2 cells were considered.

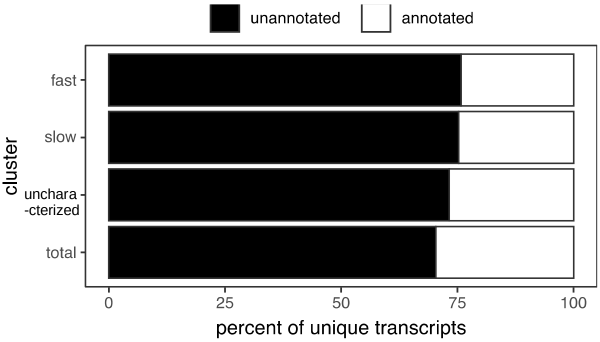

Supplementary Figure 4. Relative proportion of annotated and unannotated transcripts. Cluster identity corresponds to postclustering affiliation.

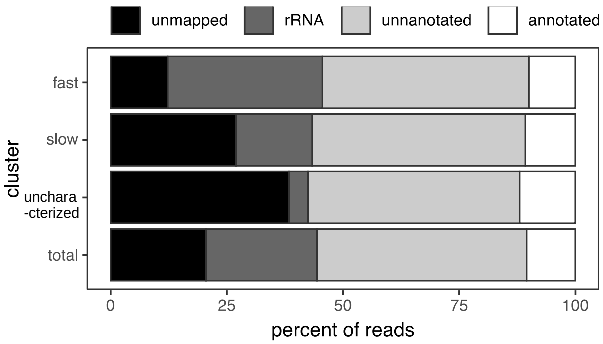

Supplementary Figure 5. Relative proportion of total number of reads that either didn't map to the assembled transcriptome, were assigned as ribosomal or mapped to either unannotated or annotated transcripts. Cluster identity corresponds to postclustering affiliation.

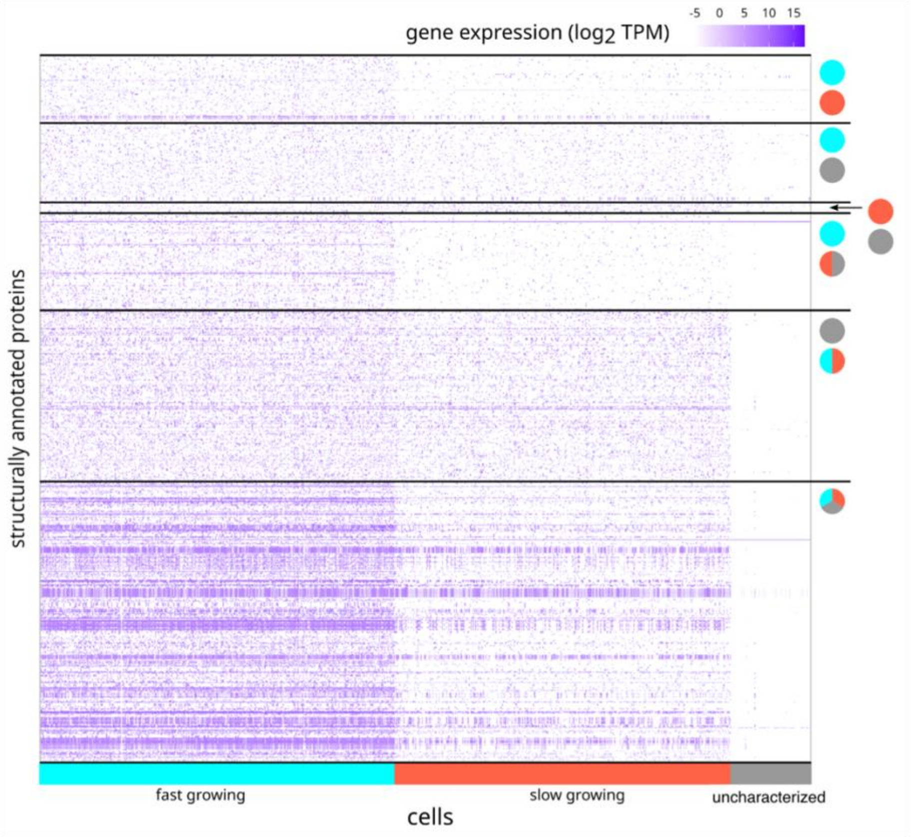

Supplementary Figure 6. Heatmap of structurally-annotated transcripts that were differentially expressed between different Ochromonas triangulata growth stages. Coloured rectangles in the horizontal axis indicate cell affiliation to clustering group. Circle pairs indicate pairwise comparisons between stages. Those cases where gene expression in one group is significantly different to that of the other two groups, the joint is represented by a split circle in which the colour of each half encodes the identity of each member. A three coloured circle represents cases in which gene expression differs significantly for any possible pairwise comparison. The colours in circles and rectangles correspond to fast-growing (cyan), slow-growing (red), and uncharacterized (grey) stages.

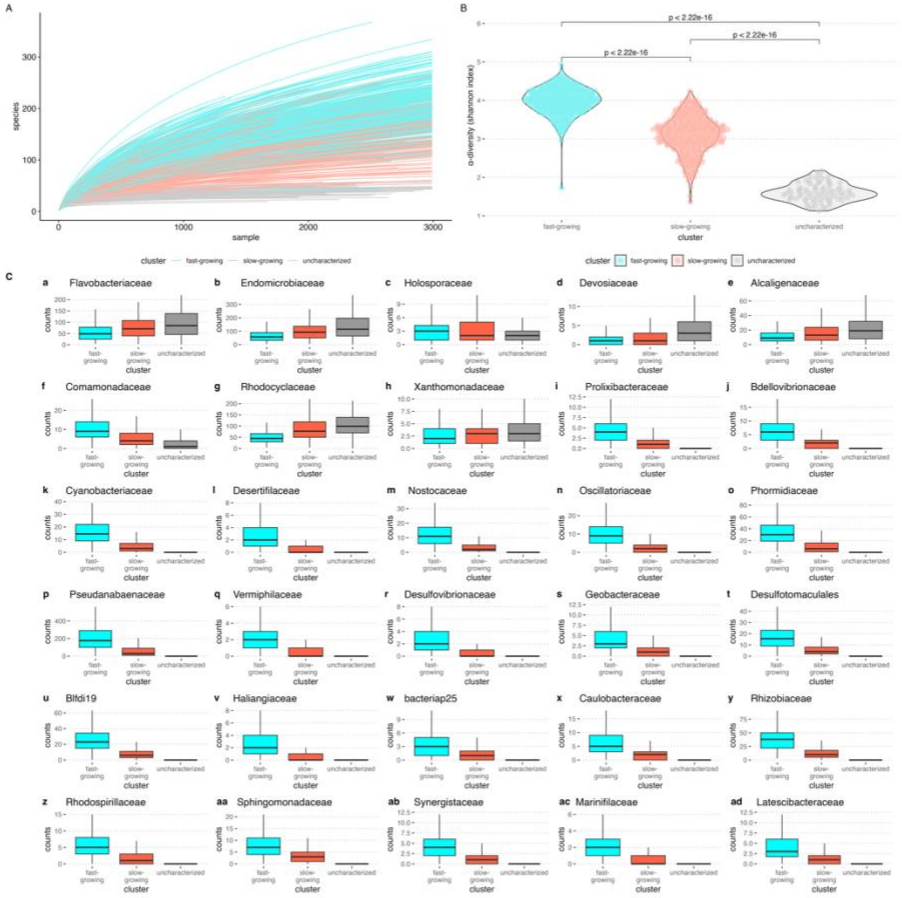

Supplementary Figure 7. Prokaryota associated with the *O. triangulata* cells in culture. (A) Rarefaction curves. (B) Alpha diversity in the three cell clusters as measured using Shannon index. P-values from pairwise wilcox tests provided. (C) Read counts of the differentially abundant families of prokaryota, identified to be significant by five tested differential abundance tests in ALDEx2, ANCOM-BC, DESeq2, LinDa, and Maaslin.

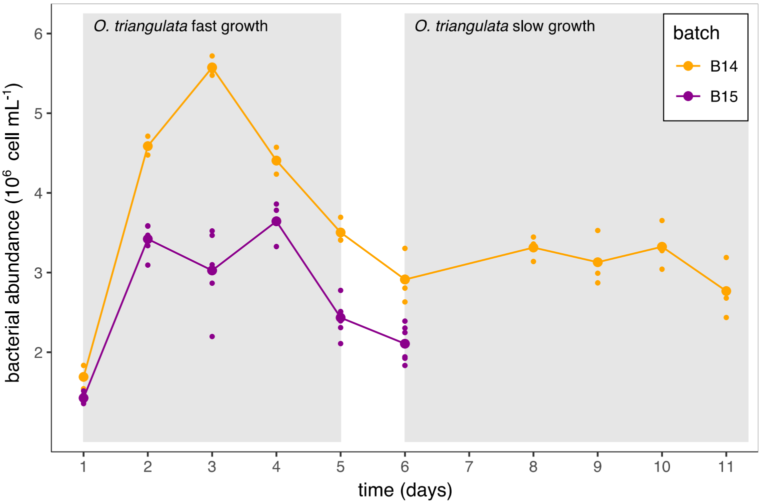

Supplementary Figure 8. Abundance of indigenous bacterial communities in co-culture with Ochromonas triangulata. Time (days) refers to the time elapsed since the last culture passing. Batch B14 was the source of the slow-growing *O. triangulata* cell consortium (sampled at day 12, not shown here), while batch B15 was the source of the fast-growing (sampled at day 2). Shaded areas indicate the concurrent fast and slow growth phases of *O. triangulata*.

Supplementary tables

Supplementary Table 1. Size distribution of the proteins in the three clusters of cells. Size refers to no. of amino acids: small (0, 200], medium (200, 400], large (400, 800].

Supplementary Table 2. Quality distribution of the proteins in the three clusters of cells. Quality refers to pLDDT scores: good (0.5, 0.7], high (0.7, 0.9], very high (0.9, 1].

Supplementary Table 3. Significantly enriched Gene Ontology: Biological Processes genesets with the pre-ranked list being ranked based on the wald statistic from the differential expression test between fast-growing and slow-growing clusters.

Supplementary Table 4. Significantly enriched Gene Ontology: Biological Processes genesets with the pre-ranked list being ranked based on the wald statistic from the differential expression test between fast-growing and uncharacterized clusters.

Supplementary Table 5. Significantly enriched Gene Ontology: Biological Processes genesets with the pre-ranked list being ranked based on the wald statistic from the differential expression test between slow-growing and uncharacterized clusters.

Supplementary Table 6. List of differentially abundant families of prokaryotes associated with the fast-growing, slow-growing, and uncharacterized populations. Column 2 provides indicates which pairwise comparison was significant for the family in column 1. Columns 3, 4, and 5 provide mean and standard deviations of the counts of the prokaryote reads associated with fast-growing, slow-growing, and uncharacterized populations respectively.

Supplementary Table 7. List of prokaryotes, agglomerated to the family taxonomic level, found associated with each sample. Counts and cluster affiliation is also presented.
